# Maternal Factors Associated with Fetal Growth and Birthweight Are Independent Determinants of Placental Weight and Exhibit Differential Effects by Fetal Sex

**DOI:** 10.1371/journal.pone.0087303

**Published:** 2014-02-06

**Authors:** Marie Cecilie Paasche Roland, Camilla M. Friis, Kristin Godang, Jens Bollerslev, Guttorm Haugen, Tore Henriksen

**Affiliations:** 1 Department of Obstetrics, Oslo University Hospital, Oslo, Norway; 2 Department of Specialized Endocrinology, Oslo University Hospital, Oslo, Norway; 3 University of Oslo, Oslo, Norway; Virgen Macarena University Hospital, School of Medicine, University of Seville, Spain

## Abstract

**Introduction:**

Maternal nutritional and metabolic factors influence the developmental environment of the fetus. Virtually any nutritional factor in the maternal blood has to pass the placental membranes to reach the fetal blood. Placental weight is a commonly used measure to summarize placental growth and function. Placental weight is an independent determinant of fetal growth and birthweight and modifies the associations between maternal metabolic factors and fetal growth. We hypothesized that maternal factors known to be related to fetal growth, newborn size and body composition are determinants of placental weight and that effects of maternal metabolic factors on placental weight differ between the genders.

**Methods:**

The STORK study is a prospective longitudinal study including 1031 healthy pregnant women of Scandinavian heritage with singleton pregnancies. Maternal determinants (parity, body mass index, gestational weight gain and fasting plasma glucose) of placental weight were explored by linear regression models, stratified by fetal sex.

**Results:**

Parity, maternal BMI, gestational weight gain and fasting glucose had positive effects on placental weight. There was a sex specific effect in these associations. Fasting glucose was significantly associated with placental weight in females but not in males.

**Conclusion:**

Maternal factors known to influence fetal growth, birthweight and neonatal body composition are determinants of placental weight. The effect of maternal factors on placental weight is influenced by sex as illustrated in the relation between maternal glucose and placental weight.

## Introduction

Maternal nutritional and metabolic conditions determine the environment in which the fetus develops [1]. Maternal metabolic markers like plasma glucose and BMI are linearly related to birthweight, risk of macrosomia (birthweight >90 percentile) and percentage body fat of the newborn, clearly demonstrated in large studies like the Hyperglycemia and Adverse Outcome Study (HAPO) [2]–[4]. We have reported similar findings in our previous work [5]–[9]. In contrast to the HAPO study we have considered placental weight as a determinant of fetal growth in addition to the maternal factors. The placenta plays a major role in fetal nutrition and fetal growth as nutrients from the maternal circulation need to be transported across the placenta to reach the fetal circulation [10]. Furthermore, the placenta itself metabolizes some of the nutrients taken up by the placenta, thereby making the placenta more than a passive conduit of nutrient transport [11]. We have previously illustrated the mediating effect of placental weight in the associations between maternal factors and three different measures of fetal growth; birthweight, intrauterine fetal growth and neonatal fat percentage [5], [7].

Placenta is an organ weighing 0.4–0.7 kilos, ranging from 447 g (SD 92) in a large study from the US [12] to 711 g (SD 156) in our Norwegian cohort [5]. The main constituent of the placenta is the villous tissue. In late pregnancy it makes up a 12–14 m^2^ physical interphase separating maternal and fetal blood [13], [14] and consists mainly of two cell layers, the syncytiotrophoblast on the maternal side and capillary endothelial cells on the fetal side [15]. Virtually any nutritional or other factor on the maternal side has to pass through the syncytio-endothelial membrane to reach the fetal blood. In addition to mediate transport of nutritional and metabolic compounds the syncytiotrophoblast works as an endocrine organ that releases a number of hormones and growth factors to the maternal circulation. These factors effectuate profound metabolic and vascular alterations in the maternal body securing adequate conditions for fetal development [16].

Placental weight is a measure commonly used to summarize placental growth and aspects of placental function. In normal pregnancy it is reasonable to assume that placental weight is related to aspects of functional capacity of the placenta [17]. The surface area of the villous tissue, a main determinant of the capacity to transport nutrients, is linearly related to placental weight [18], as is maternal plasma levels of placental hormones [19]. Accordingly, factors that determine placental weight may also be important determinants of functional capacities of placenta and fetal nutritional conditions. Placental size can be affected by changes in maternal nutrition. Data from pregnancies during the Dutch famine showed that placental area was reduced [20]. In the second and third trimester of pregnancy the changes in maternal diet in the month of Ramadan were associated with reduced placental weight [21].

There are sex differences in weight and body composition at birth [22], differences that may have impact on the future health of the newborn. Sex differences have also been reported in placental size. In the Dutch famine placental area among boys were reduced, but less so in girls [23]. A similar trend was observed in the Ramadan study [21].

In the present study, we hypothesized that maternal factors known to be related to fetal growth, newborn size and body composition are determinants of placental weight. Based on previous observations of ours, we also hypothesized that the effects of maternal metabolic factors on placental weight differ between the genders.

### Aim of the study

To study the effects of maternal factors (parity, BMI, gestational weight gain and fasting glucose) on placental weight, stratified by fetal sex.

## Materials and Methods

### Ethics

The study was approved by the Regional Ethics Committee for medical research South-East (S-01191). All participants signed a written informed consent.

The STORK study is a prospective cohort study with a longitudinal design including 1031 healthy women of Scandinavian heritage with singleton pregnancies who gave birth at Oslo University Hospital, Rikshospitalet between 2002 and 2008. Exclusion criteria were multiple pregnancies, known pre-gestational diabetes and severe chronic diseases (lung, cardiac, gastrointestinal or renal), and pregnancies with fetal malformations discovered at the routine scan in week 18 of pregnancy. Only 37 were lost to follow up, mostly because some participants moved to a different area during pregnancy and some accidentally delivered at a different hospital. Details about the inclusion of patients have been published [5].

Each pregnant woman had four antenatal visits, scheduled at weeks 14–16 (visit 1), weeks 22–24 (visit 2), weeks 30–32 (visit 3) and weeks 36–38 (visit 4). Clinical data and blood samples were collected at each visit. Parity was coded as P0 for nulliparous women and P1 for those with one or more previous births. BMI (kg/m^2^) was calculated by height and weight at the first visit. Weight was measured by a calibrated scale at each visit. Gestational weight gain (GWG) was calculated as both the total GWG (difference between weights measured at visit 4 and visit 1) and weight gain between each visit. Measured weight at the first visit was used instead of pre pregnancy weight to avoid recall bias.

Blood samples were drawn in the morning after an overnight fast. Fasting plasma glucose was measured at visit 1 (weeks 14–16) and visit 3 (weeks 30–32) in EDTA blood by the Accu-Check Sensor glucometer (Roche Diagnostics GmbH, Mannheim, Germany). Due to an unexpected increasing trend in fasting plasma glucose over time, the glucose values were statistically adjusted [24]. Placental weight including cord and membranes was measured by the midwife within one hour after the delivery.

### Statistical methods

Descriptive data are reported as mean and standard deviations or numbers and percentage as appropriate and are shown in Table 1 and 2. The outcome variable was placental weight (g). The maternal characteristics chosen as independent variables were parity, BMI measured at visit 1 (weeks 14–16), total GWG (between visit 1 and visit 4), GWG between each visit and fasting plasma glucose at visit 1 and 3. There was no multicollinearity between the variables. All analyses were adjusted for gestational age since placental weight depends on gestational age. The associations between maternal characteristics and placental weight were analyzed by univariate and multiple linear regression models.

**Table 1:** Characteristics of the mothers.

	n	%	Mean	SD	Range
Age (years)			31.3	3.9	19–42
Married or partnership	1011	98.1			
Higher education ≥15 years	885	85.8			
Para 0	545	52.9			
Daily smoking during pregnancy	28	2.7			
BMI visit 1			24.5	3.9	17.2–43.9
Gestational Weight Gain (kg) v1–v4			10.6	3.5	−1.2–29.4
GWG (kg) v1–v2			3.6	1.8	−3.2–10.8
GWG (kg) v2–v3			4.1	1.8	−6.9–12.5
GWG (kg) v3–v4			2.9	1.9	−4.8–10.7
Fasting plasma glucose v1 (mmol/l)			4.0	0.39	2.6–5.3
Fasting plasma glucose v3 (mmol/l)			4.1	0.45	2.9–6.2
Gestational diabetes *	56	5.5			
Preeclampsia #	39	3.8			

*according to WHO-criteria; ≥7.8 mmol/l 2 hours after 75 g glucose.

# BP>140/90 and proteinuria.

**Table 2:** Characteristics of the newborns.

	Total		Boys		Girls		
	n = 1031		n = 549		n = 482		
	Mean	(SD)	Mean	(SD)	Mean	(SD)	p-value*
Gestational age at birth (weeks)	39.5	(1.8)	39.6	(1.9)	39.3	(1.9)	0.014
Birthweight (g)	3588	(574)	3667	(576)	3494	(562)	<0.001
Length (cm)	50.8	(2.3)	51.3	(2.3)	50.3	(2.2)	<0.001
Head circumference (cm)	35.0	(1.6)	35.4	(1.5)	34.6	(1.6)	<0.001
Placental weight (g)	711	(156)	715	(153)	707	(160)	0.4
Fetal/placental ratio	5.17	(0.9)	5.26		5.08	(0.8)	0.001

*difference between boys and girls, independent sample t-test.

First we estimated the effects of parity, BMI, GWG and fasting glucose on placental weight by linear regression (Table 3). This analysis was then repeated, stratified by fetal sex (Table 4 and 5). Fasting glucose at visit 1 and visit 3 were correlated (r = 0.46, p<0.001). We included only one measurement of fasting glucose in the multiple model at the time. Only fasting glucose at visit 3 remained statistically significant and was included in the final model.

**Table 3:** Effects of maternal characteristics on placental weight (g) in the total cohort (n = 1031).

	Univariate			Multiple		
Variable	Unadjusted B	95% CI	p-value	Adjusted B	95% CI	p-value
BMI	7.9	5.5–10.3	<0.001	6.7	4.1–9.3	<0.001
Total GWG v1–v4 (kg)	5.2	2.3–8.0	<0.001	5.6	2.8–8.4	<0.001
GWG v1–v2 (kg)	11.2	5.5–17.0	<0.001			
GWG v2–v3 (kg)	4.4	−1.1–9.9	0.11			
GWG v3–v4 (kg)	4.6	−0.7–10.0	0.09			
Parity (P1 vs. P0)	37.9	18.6–57.1	<0.001	29.2	10.1–48.4	0.003
Fasting glucose v1*(mmol/l)	3.8	9.1–58.6	0.007			
Fasting glucose v3*(mmol/l)	55.3	34.0–77.0	<0.001	32.9	10.0–55.7	0.005
Gestational age (weeks)	26.7	21.6–31.9	<0.001	18.9	11.6–26.1	<0.001

Results from univariate and multiple linear regression models.

*Fasting glucose at visit 1 and fasting glucose at visit 3 were entered separately in the multiple models, but only fasting glucose at visit 3 remained statistically significant and was entered in the final model presented in Table 3.

**Table 4:** Effect of maternal factors on placental weight (g) for boys (n = 549).

	Univariate			Multiple		
Variable	Unadjusted B	95% CI	p-value	Adjusted B	95% CI	p-value
BMI	7.0	3.8–10.3	<0.001	7.3	3.8–10.7	<0.001
GWG v1–v4 (kg)	4.5	0.7–8.4	0.022	4.6	0.8–8.5	0.019
Parity (P1 vs. P0)	23.1	−2.7–49.0	0.079	18.4	−7.4–44.3	0.16
Fasting glucose v1(mmol/l)	9.9	−23.6–43.4	0.56			
Fasting glucose v3(mmol/l)	37.7	7.5–67.8	0.015	17.8	−13.9–49.4	0.27
Gestational age (weeks)	27.0	20.3–33.6	<0.001	17.1	7.0–27.1	0.001

Results from univariate and multiple linear regression models.

**Table 5:** Effect of maternal factors on placental weight (g) for girls (n = 482).

	Univariate			Multiple		
Variable	Unadjusted B	95% CI	p-value	Adjusted B	95% CI	p-value
BMI	9.1	5.4–12.8	<0.001	6.3	2.4–10.3	0.002
GWG v1–v4 (kg)	5.9	1.7–10.1	0.006	6.4	2.4–10.7	0.003
Parity (P1 vs. P0)	53.4	24.7–82.0	<0.001	39.6	10.6–68.8	0.008
Fasting glucose v1* (mmol/l)	59.9	23.2–96.6	0.001			
Fasting glucose v3* (mmol/l)	73.6	43.1–104.2	<0.001	44.2	11.3–78.8	0.011
Gestational age (weeks)	26.1	18.4–33.7	<0.001	21.2	9.6–30.9	<0.001

Results from univariate and multiple linear regression models.

*Fasting glucose at visit 1 and fasting glucose at visit 3 were entered separately in the multiple models, but only fasting glucose at visit 3 remained statistically significant and was entered in the final model presented in Table 5.

Variables with a p-value <0.10 in the univariate analyses were included in the multiple models. A p–value <0.05 was considered statistically significant.

SPSS 18.0 for Windows (SPSS Inc., Chicago, IL) was used for all analyses.

## Results

The mean birthweight was 3588 g (SD 574) and mean gestational age at birth was 39.5 (SD 1.8) weeks. Mean placental weight was 711 g (SD 156). Only 2.7% smoked during pregnancy.

Boys were heavier (3667 g vs. 3494, p<0.001) and longer (51.3 cm vs. 50.3, p<0.001) than girls. Boys also had a higher fetal: placental ratio than girls (5.26 vs. 5.08, p<0.001). These differences remained statistically different when corrected for gestational age. There were no statistically significant differences between mothers of boys and mothers of girls in parity, maternal BMI, GWG or fasting glucose (0.8<p<0.9).

Several maternal characteristics had a positive effect on placental weight, including parity, maternal BMI, GWG and fasting glucose (Table 3). Adjusted estimates showed that multipara had nearly 30 g heavier placentas than primipara, one unit higher BMI gave 6.7 g heavier placenta and one kilo increase in GWG gave 5.6 g heavier placenta. GWG had an independent positive effect on placental weight. GWG in the first half of pregnancy (weight at visit 2 minus weight at visit 1) was significantly associated with placental weight (p<0.001), whereas weight gain later in pregnancy was not associated with placental weight (Table 3).

In the multiple models fasting glucose at visit 3 was significantly associated with placental weight in females but not in males. Fasting glucose at visit 1 was significant in univariate analyses, but was non-significant in the multiple models for the total cohort as well as for girls and boys separately. In the final models, we therefore included fasting glucose measured at visit 3.

## Discussion

In the present study, we found that parity, BMI, GWG, fasting glucose at visit 3 and gestational age had positive, independent effects on placental weight. Our study showed that an increase of one unit of BMI increased placental weight by 7.9 g (unadjusted). The linear association between increasing BMI in categories and placental weight was convincingly illustrated by Wallace et al. in a material from Aberdeen including 55 105 placentas from 37 842 women [25]. Our effect estimates were based on BMI as a continuous variable. By categorizing our data, we found a similar linear association (data not shown). A Dutch study on determinants of placental weight reported an effect of pre-pregnancy BMI of 5.9 g pr unit BMI [26].

Given the current finding that BMI influences placental weight, our results are compatible with the concept that some of the effect of BMI on birthweight is mediated through a promotion of placental growth. Apart from glucose, there are possibly other biological factors related to BMI that exert an effect on placental growth. Which factors are still to be elucidated. However, we recently reported that maternal plasma total cholesterol was positively associated with placental weight, whereas HDL cholesterol had a negative effect [7].

Multipara in our study had 37.9 g higher placental weight than primipara. Data from the Aberdeen study referred to above was used to create placental weight charts specific for gestational age, gender and parity. This study showed that the difference between boys and girls in placental weight was approximately 15 g regardless of parity, whereas the effect of parity was larger, approximately 27 g for both girls and boys [27].

Total GWG was restricted to the difference between weight measured at visit 4 and weight measured at visit 1. Pre-pregnancy weight could have introduced a recall bias that generally is difficult to estimate and we did not have complete data on weight measured at delivery. Our results showed that it was GWG between visit 1 and visit 2 that was significantly associated with placental weight, whereas GWG after visit 2 did not show a significant association. Hence, GWG in early pregnancy had a positive effect on placental weight. This finding is in accordance with a study by Diouf and coworkers who found that weight gain in the first trimester was positively associated with birthweight. Path analysis indicated that this was not a direct effect of weight gain on birthweight, but rather an effect mediated by placental weight [28]. Maternal weight gain in early pregnancy is a placental hormone driven increase in plasma volume and fat stores making the association between early GWG and placental mass biologically plausible.

Fasting glucose at visit 3 was positively associated with placental weight in the total cohort, but when stratified by fetal sex the effect was only significant for the females. Previous results from our group showed that fasting glucose was associated with birthweight in girls, not in boys [9]. Thus, glucose has an effect on both placental weight and birthweight only among girls. Comparable studies in humans on the effect of maternal glucose in early pregnancy on placental weight in normal pregnancies are few. However, in pregnant women diagnosed with diabetes type 1, elevated hemoglobin A_1c_ in first trimester was associated with increased birthweight and placental weight as well as increased GLUT 1 expression compared to normal pregnancies [29]. In pregnant rats, administration of three injections of glucose in early pregnancy resulted in an increase not only in birthweight, but also in the placental weight by 10% [30]. Taken together, this suggests that early placental growth is sensitive to maternal glucose levels and that the increased birthweight might be mediated through growth of the placenta. We found effect of fasting glucose at visit 1 only in the univariate analyses.

In our study boys were heavier and had a higher fetal: placental ratio than girls, indicating that boys have more efficient placentas than girls. Ericsson et al also observed that boys tended to be longer and have larger head circumferences at birth for any placental weight. They suggested that boys invest in brain growth rather than placental growth, which leaves them with less reserve capacity and makes them more vulnerable to under nutrition [31].

As mentioned above, the effect of fasting glucose on placental weight was only significant for female placentas, not male placentas. Epidemiological studies have shown sex specific differences in fetal and neonatal mortality and morbidity. A study from the Birth Registry of Norway including more than 1.800.000 births showed that the male/female ratio was higher among preterm deliveries, particularly in the lower gestational age groups [32]. Even when taking account of the number of boys born preterm is higher than girls, male fetuses are still more likely to have poor outcomes compared to females [33]. The increased vulnerability described among boys in general can be attributed to the male gender, keeping in mind that the male fetoplacental unit also includes the male placenta. Recently sex specific differences in the placenta have been identified in terms of gene expression, histological examination and cytokine production [34]. There are sex specific differences in placental gene expression that are not limited just to X and Y linked genes, but also to genes related to immune pathways [35]. Challis et al. suggested that sex differences in placental responses may contribute to the increased incidence of preterm delivery observed for male fetuses [36].

Fasting glucose is one aspect of maternal metabolic status whose effect on placental weight was sex specific in our study. Our results showing an effect of fasting glucose on placental weight in female placentas only adds to the emerging understanding of the physiology linked to sex specific effects on the placenta.

### Strengths and limitations

We present a large prospective, longitudinal study with 1031 pregnancies in which maternal factors were well characterized. Limitations include lack of reliable data on pre-pregnancy BMI. To avoid recall bias we therefore calculated BMI based on measured height and weight at the first visit. We have no estimation of placental weight by ultrasound to assess growth of the placenta longitudinally and have therefore used placental weight after delivery as our outcome variable. The placentas were not trimmed, but weighed including the umbilical cord and membranes. However, a strong correlation (r = 0.98) has been reported between trimmed and non-trimmed placentas [37].

## Conclusions

Our data support the hypothesis that maternal factors known to influence fetal growth, birthweight and neonatal body composition also are determinants of placental weight. The differential effect of maternal factors on placental weight by fetal sex was illustrated in the relation between maternal glucose and placental weight.
